# Contributions to the Practice of Midwifery

**Published:** 1845-12

**Authors:** J. Hall Davis

**Affiliations:** Physician to the Royal Maternity Charity


*Contributions to the Practice of Midwifery.* By J. HALL DAVIS, M.D., Physician to the Royal Maternity Charity. Uterine hæmorrhage in the sixth and seventh months of pregnancy accidental and unavoidable.—Treatment, with some remarks on the indications for the use of the "plug."

May 9th, 1844.—My attendance was required in the case of Mrs. G——, aged thirty-two, residing in my immediate neighborhood, previously in good health, pregnant of her third child, and six months advanced in her gestation. Late on the previous evening she had been taken with flooding, which she supposed had been caused by a fright, having been nearly run over by a horse. Early on the following morning, after the escape of some coagula of blood, the hæmorrhage had ceased. I found her at my visit, at midday, as follows:—Pallid, having prior to the occurrence been of good colour; pulse quick, sharp, but regular; extremities cold, not clammy; sense of throbbing in the head, with giddiness, increased by the upright posture; she had felt faint, but had not decidedly fainted; had had no pain in the back, had experienced slight painful sensations in the lower part of the hypogastrinum, but none of a bearing-down character. I directed her to be kept exceedingly quiet in bed, the room to be kept cool, all drinks to be taken cool; nothing in the way of food, but a little weak broth, farinaceous diet, and no stimulating drinks. Fifteen minims of dilute sulphuric acid in water every four hours. This plan—with the exception that she was allowed, the hæmorrhage not returning, to leave her bed at the end of two days, maintaining still the horizontal posture—was continued for about a week, when she had sufficiently recovered to walk out a little. She was, however, cautioned against incurring fatigue, lifting heavy weights, or making any strong exertion.

June 21.—At two, A. M., the hæmorrhage returned, without any apparent cause, but not copiously, and I was requested by Mr. Browne, her attendant, to see her. My visit was made at a quarter past twelve. On examination, per vaginam, I found the orifice of the uterus closed, the nipple-like projection of the lower segment of the uterus was evident, corresponding to the period of her gestation, which was about six weeks short of the full term. The head of the child could not be felt through the anterior walls of the uterus behind the pubes, consequently the ballottement could not be practised: the reason of this became apparent subsequently. The hæmorrhage was moderate, and had not produced even faintness. She had completely recovered from her first attack in

May. She had had no pain; the pulse of good volume and strength. I suggested to my friend, for the present, a repetition of the same measures and precautions, precisely as before, and the expediency of his watching his patient closely.

On the afternoon of the following day the hæmorrhage became profuse, and the patient extremely faint, her face pallid, the pulse small; when, being sent for, I immediately determined, as the hæmorrhage continued, upon the use of the plug. A broad bandage was at the same time applied firmly around the abdomen. She was desired to remain quiet upon the bed, was allowed a little beef tea from time to time, and was visited at intervals. At half-past eight in the evening, there having been active pains during the previous two hours, the plug was removed. The hæmorrhage had been effectually suppressed. The mouth of the womb was now dilated to the diameter of two fingers' breadth, but admitted not of further extension, to enable the hand to be introduced. At intervals of ten minutes and a quarter of an hour the pains were repeated, the membranes becoming tense during their presence, and projecting slightly through the uterine orifice. An edge of the placenta could be felt in front of the head, on passing the finger up, in the absence of pain. At half-past one, A.M., the pains having very much slackened in force, some ergot of rye was exhibited, which had the desired effect of increasing their strength, but the flooding returned to a considerable amount. Upon being sent for, and finding the orifice of the uterus not perceptibly more dilated than at my former visit, and not dilatable, I now ruptured the membranes, and discharged the liquor amnii, hoping thus to put a stop to the flooding, and not wishing again to put her to the inconvenience inseparable from the use of the plug. The hœmorrhage was thus arrested for an hour, and then returned, and she again became faint and pallid. The orifice of the uterus being now only so far dilated as to admit of the introduction of the tips of three fingers, disposed conically, with considerable difficulty, I was compelled to return to the use of the plug, and applied the bandage to the abdomen a little more firmly, and now prescribed an opiate in a full dose, with the view, if possible, of relaxing the rigidity of the uterine orifice, and of procuring her some refreshing sleep.

Visit, ten, A.M.—She had had three hours sleep, and felt greatly better. The plug last employed, in the absence of sponge, consisted of pellets of old linen; it had permitted of a very slight return of hæmorrhage; nevertheless, her pulse was softer and fuller, the extremities warm, and the faintness had ceased; the pains had subsided; the urine was drawn off by the catheter, and the plug was made more efficient by the introduction of some pieces of sponge. At one P.M., she had had some trifling grinding pains in the small of the back, but none of a bearing-down character. the plug was therefore allowed to remain; there had been no oozing of blood since the last visit. At nine P.M., the same kind of pains had occurred at intervals, but none of an expulsive kind. Being wearied by want of rest,, and the teasing character of the pains, another full opiate was exhibited, and she was left with the plug in the vagina, as a safeguard against return of hæmorrhage. At one A.M., Mr. Browne was sent for. She had had three hours' refreshing sleep, and had been awakened by the pains, which were now strongly bearing. The plug was removed, and a still-born, blanched, and premature child, was quickly expelled, with the chord twisted around its neck.

The placenta was thrown off by the action of the uterus into the vagina, without hæmorrhage, at the end of forty minutes, and thence removed; the bandage previously round the abdomen was tightened, and the uterus was left firmly contracted.

July 11th.—The patient was sufficiently recovered to walk out without risk, having improved steadily under the judicious care of my friend Mr. Browne.

*Remarks.*—As soon as the mouth of the uterus was sufficiently dilated to admit the finger within it, the lower edge of the placenta was distinctly felt in front of the membranes of the ovum, and in attempting to pass the finger higher up, between them and the walling of the uterus, I was prevented from doing so by the placental attachment. That portion of the membranes which presented at the mouth of the womb was uncovered by placental tissue, and through it the head was found to be the presenting part. The head of the child had not previously been detected, nor, indeed, could it now, through the uterine walling, by the ordinary vaginal examination; nor, consequently, had it been practicable to practise the "ballottement," as in cases of normal pregnancy, by reason of the intervening attachment of the placenta to the cervix.

This patient sustained a hæmorrhage, in the first instance, in the sixth month of pregnancy, from an obvious and accidental cause—a fright. She recovered from this at the end of a week, so as to be able to walk out, and we hoped there would be no return of it. On the 21st of June, six weeks subsequently, the hæmorrhage recurred, without any obvious cause, which led me to suspect it to be of the unavoidable kind, the result of "placenta prævia," which proved to be the case.

I had recourse to the "plug," preferably to discharging the liquor amnii in the first instance—a practice which I have pursued with success on many occasions,—that the advantage afforded by the bag of waters entire, in dilating the uterine orifice, might be retained, and to facilitate the operation of turning, should that proceeding become necessary. The material of the "plug" which I employed, and which I consider preferable, by far, to any other, was sponge; introduced in separate pellets, until the vagina was completely filled from its uterine extremity to its outlet, a measure answering the two-fold object of stopping the flooding, and, by its irritation, inducing the action of labor; a broad bandage was at the same time applied to the abdomen, so as to afford an uniform support to the walls of the uterus. The patient was thus placed in a state of perfect security against hæmorrhage, until the pains of parturition should be instituted; indeed, until it might be presumed, from the continuance of the pains, and their character, that the orifice of the uterus had dilated sufficiently for the safe passage of the hand into the uterus, and performance of the operation of turning, or for the ready transit of the child by its presentation.

It will be seen that I subsequently, to put a stop to the flooding, which had returned after the removal of the first plug, discharged the liquor amnii by rupturing the membranes, to save the patient the inconvenience of a repetition of the plugging, and that, in consequence, the hæmorrhage ceased, but returned at the end of an hour, so that the former means was not efficient, as was the latter, in permanently suppressing the hæmorrhage.

Some practitioners have, and, agreeably to my experience, without foundation, dreaded the possibility of internal hæmorrhage occurring under the practice of plugging the vagina during pregnancy, or of its masking an internal hæmorrhage, which might indeed, possibly happen, were the body of the uterus relaxed so as to admit of an expansion of its cavity; but the orifice of the uterus is supposed to be in a state of rigidity when the plug is employed, and we may presume that the entire parietes of the organ must be in a somewhat similar condition, or, at any rate, so far in possession of their natural tonicity as not to admit of unlimited development; but at all events the application of a broad belly-band, with the ordinary precautionary measures, to be adopted in cases of hæmorrhage, would remove all the chances of such an event occurring—it has never once happened under my cognizance.

On the other hand, should the plug not be had recourse to, in a case in which the liquor amnii has been discharged, the hæmorrhage continues in considerable quantity, and as the hand cannot be passed through the uterine orifice to turn the child, without inflicting contusion or laceration, the patient must inevitably die from loss of blood, the victim of an unfonuded dread.—*London Lancet.*

